# 

**Published:** 1894-03-03

**Authors:** 


CONTENTS.

Medical Ideals Unrealised - 385

Around the Hospitals - 386

Studies in Applied Sociology. IV.—Scientific Temperance - 388

Medical Progress and Hospital Clinics—

Some General Considerations as to the Various Methods of Treatment to be Adopted in Skin Affections - 393

Painful Menstruation and its Treatment - 394

MEDICAL PROGRESS.—Nerve Disease - 395

Surgery of the Genito-Urinary Organs - 397

Diseases of the Tonsils - 398

NEW DRUGS AND PREPARATIONS.—Perchloride of Mercury—Methylacetanilide - 399

NEW APPLIANCES AND THINGS MEDICAL - 399

Diuretin and Diuretics—Practical. By Sir BENJAMIN WARD RICHARDSON, M.D., F.R.S. - 387

Modern Aspects of Pernicious Anæmia.—II - 389

Practical Aspects of Medical Science - 390

Annotations.—Massage in Edinburgh—The Net Gain of the Chloroform Commission—Aids to Longevity - 391

Notes and News - 392

Editor's Letter Box - 400

The Institutional Workshop—

WITHIN THE HOSPITALS.—Royal Infirmary, Bristol - 401

PRACTICAL DEPARTMENTS - 403

HOSPITAL FESTIVALS, MEETINGS, &c. - 403

The Book World of Medicine and Science - 405

EXTRA SUPPLEMENT.—THE NURSING MIRROR.

News from the Nursing World.—The Princess May's Ward—Probationers in Charge—Workhouse Nursing Association—Profitable Patchwork—Healthy Ely—Queen's Nurses at Gateshead—A Qualified Nurse—Nurses at Birmingham—Liberality at Londonderry—Free Passes for Nurses—Queen's Nurses in Ireland—Only Her Due—India—Charges at Addenbrooke's—Short Items - ccxi
On General Nursing. I.—Measles - ccxiii
The Beginnings of Insanity - ccxiv
Lectures at St. George's Hospital.—History of the Hospital (*continued*) - ccxv
Women's Work.—High School Teachers - ccxvi
Novelties for Nurses—Minor Appointments - ccxvi
Health Resorts for Consumptive Patients - ccxvii
Our Melbourne Letter—Notes and Queries - ccxviii
For Reading to the Sick - ccxviii
The Muses' Looking Glass.—Bona-fide Facts About Bones - ccxix
Everybody's Opinion - ccxx

